# Simplified, automated methods for assessing pixel intensities of fluorescently-tagged drugs in cells

**DOI:** 10.1371/journal.pone.0206628

**Published:** 2018-11-01

**Authors:** Allan Kachelmeier, Tsering Shola, William B. Meier, Anastasiya Johnson, Meiyan Jiang, Peter S. Steyger

**Affiliations:** 1 Oregon Hearing Research Center, Oregon Health & Science University, Portland, Oregon, United States of America; 2 National Center for Rehabilitative Auditory Research, VA Portland Health Care System, Portland, Oregon, United States of America; Pennsylvania State Hershey College of Medicine, UNITED STATES

## Abstract

Assessing the cytoplasmic uptake of fluorescently-tagged drugs in heterogeneous cell types currently involves time-consuming manual segmentation of confocal microscopy images. We developed a set of methods that incorporate map algebra techniques to facilitate and expedite image segmentation, particularly of the parenchyma of intermediate cells in the stria vascularis of the inner ear. Map algebra is used to apply a convolution kernel to pixel neighborhoods to create a masking image to select pixels in the original image for further operations. Here, we describe the utility of integrated intensity-based, percentile-based, and local autocorrelation-based methods to automate segmentation of images into putative morphological regions for pixel intensity analysis. Integrated intensity-based methods are variants of watershed segmentation tools that determine morphological boundaries from rates of change in integrated pixel intensity. Percentile- and local autocorrelation-based methods evolved out of the process of developing map algebra- and integrated intensity-based tools. We identified several simplifications that are surprisingly effective for image segmentation and pixel intensity analysis. These methods were empirically validated on three levels: first, the algorithms were developed based on iterations of inspected results; second, algorithms were tested for various types of robustness; and third, developed algorithms were validated against results from manually-segmented images. We conclude the key to automated segmentation is supervision of output data.

## Introduction

“*Full automation of pattern recognition slipped away time and again*.” Peter Galison, Image and Logic [1]

**Fig 1:**
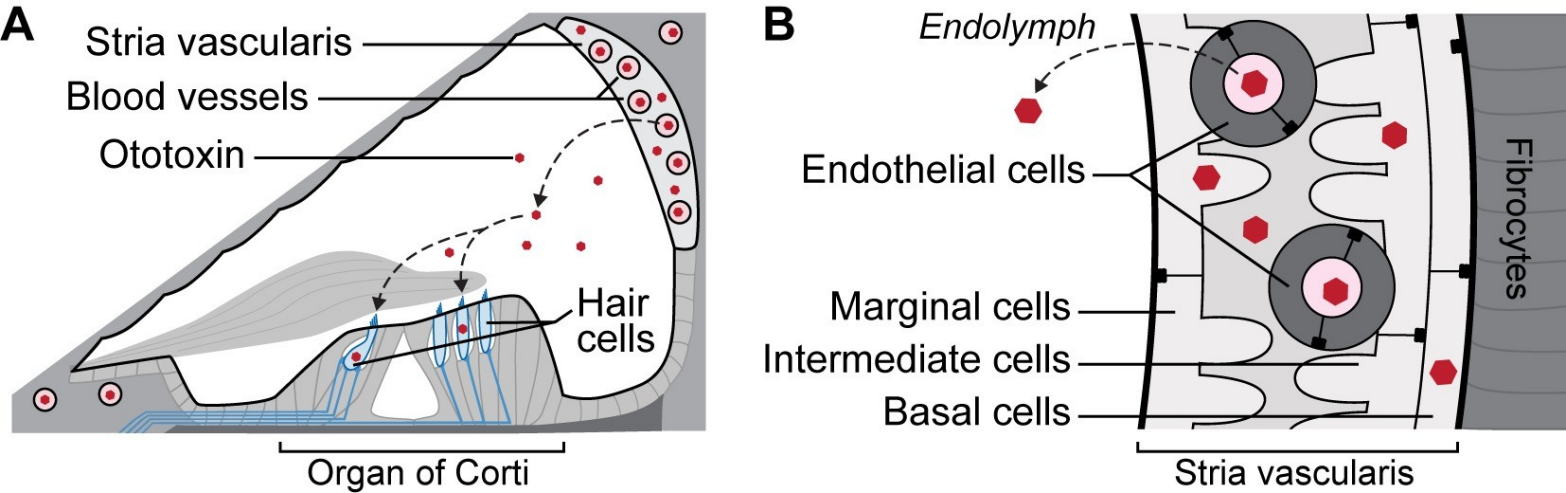
Schematic diagrams of the mammalian cochlea. **(A)** A cross-sectional diagram of the cochlear duct showing the organ of Corti containing the sensory hair cells resting on the basilar membrane (dark grey), and the highly-vascularized stria vascularis on the lateral wall of the cochlea. Circulating ototoxins, like cisplatin and aminoglycosides, preferentially cross the blood-labyrinth barrier in the stria vascularis, and are cleared into endolymph prior to entering hair cells across the apical surface of hair cells. **(B)** A higher resolution diagram of the stria vascularis, showing the relative positions of marginal, intermediate and basal cells in the stria vascularis, and the adjacent fibrocytes in the perilymphatic domain of the cochlea. Circulating ototoxins are trafficked across the endothelial cells and into marginal cells prior to clearance into endolymph, while also entering other cells within the stria vascularis. Diagrams are not to scale.

Gentamicin is an ototoxic aminoglycoside antibiotic that is readily trafficked from the bloodstream across the blood-labyrinth barrier into the inner ear, prior to clearance into inner ear fluids (primarily endolymph; Fig 1A) and uptake by inner ear sensory hair cells [2]. Once in hair cells, these drugs exert their ototoxic effect that can lead to permanent hearing loss, profound deafness as well as vestibular deficits [3, 4]. The intra-cochlear trafficking of systemically-administered aminoglycosides was identified by tracking fluorescently-tagged gentamicin predominantly through the stria vascularis (endothelial, basal, intermediate, and marginal cells) [5]. This pathway was validated manually by semi-quantification of pixel intensity for fluorescently-tagged gentamicin in the cytoplasm of heterogeneous cell types in the cochlear lateral wall [2, 6, 7] (Fig 1B).

Until recently, pixel intensity analysis of confocal images required manual segmentation (separation of the image into discrete sections) to isolate salient features (or remove inappropriate cells or artefacts) using digital erasers in ImageJ or Photoshop. However, hand-segmentation is tedious and time-consuming, particularly when segmenting complex structures such as branching blood vessels in the highly-vascularized stria vascularis (Fig 1B). Considerable effort and time would be saved if automated batch processing of confocal images to remove the brightly-fluorescent blood vessels were available. Fully-automated segmentation of confocal images, totally eliminating the user, remains unlikely as attempts at stand-alone segmentation (or pattern recognition) have not been fully successful [1]. Consequently, we reasoned it would be better to develop segmentation tools that incorporate some degree of user input (supervision), largely in the form of choosing parameters and applying appropriate algorithms, and then verifying the results. Thus, we focused on developing machine segmentation with user supervision to qualitatively improve upon time-consuming manual segmentation. The object was to validate machine segmentation of fluorescently-tagged gentamicin in the cytoplasm of heterogeneous cell types in confocal microscopy images.

Meijering [8] reviewed several methods of cell segmentation, including feature detection, morphological filtering, region accumulation, deformable model fitting, and noted that most segmentation is intensity thresholding. In this method, the pixel intensity of the region of interest is distinguished from its field as above or below a specific pixel threshold. However, the basis for thresholding need not be restricted to pixel intensity. For example, local autocorrelation methods are useful for identifying basins of homogeneity in local pixel neighborhoods. These threshold-based methods contrast with those that focus on detecting edges and gradients. The rate of change in pixel intensities defines the gradients. In morphological filtering, image pixels are transformed by a function to create a composite mask used to select out original pixels for further analysis, essentially a reductive procedure. The region accumulation approach grows a region of interest until it loses a boundary with a contiguous expanding region. This is a bottom-up method that identifies a region or neighborhood of pixels by expanding a global property, e.g., watershed segmentation. Deformable model fitting generalizes watershed segmentation, with features defined by level sets (isocontours) in local energy space [8].

We developed our initial ideas from observing that morphology in our images is mirrored in differential pixel intensities, suggesting that feature detection or region accumulation methods would be effective. We also noted local characteristics can be stored in masking images using a map algebra focal function (by calculating new pixel values as a function of neighboring pixel values). Map algebra is a set of techniques for performing operations on images, primarily used in geographical information systems (GIS) and epidemiology [9]. Given morphology is reflected in the rate at which pixel intensity is changing, the masking image gives opportunity to store temporary values and make image calculations. Map algebra is important for both smoothing the images and providing a means of communication between the identical coordinates of the original image and masking image. This smoothing enables effective elimination of anomalous pixels from subsequent statistical analyses. Correspondingly, our early algorithms combined detection of the rate of change in integral pixel intensity with local smoothing facilitated by the map algebra.

Here, we describe the utility of integrated intensity-based, percentile-based, and local autocorrelation-based methods to automate segmentation of confocal microscopy images into putative morphological regions for pixel intensity analysis for statistical analyses between experimental groups as in references [2, 6, 7]. The resulting automated tools, in combination with user insight, facilitates semi-quantitative assessment of pixel intensity in confocal images. User input includes supervision in choosing the appropriate segmentation algorithm and setting parameters, and supervision to oversee the result. Although these automated methods are designed to speed up semi-quantitative assessment of mean pixel intensity for fluorescently-tagged gentamicin in confocal microscopy images, they are general enough to segment other types of microscopy data, especially for images that can be segmented into three regions. We subsequently discuss the assumptions and robustness of machine segmentation, its advantages and limitations, and we contrast these with manual segmentation.

## Methods

The segmentation code was written in R [10], a statistical environment well-developed for spatial statistics, utilizing tools available within the R statistical environment, including facilities for upload and display of images. However, the general approaches we used for segmentation could easily be implemented in other scripting languages such as MATLAB or Mathematica. All images were obtained from confocal microscopy of tissues collected for hypotheses addressed in other published studies, for example [7]. The care and use of all preclinical models in this study were approved by the Animal Care and Use Committee of Oregon Health & Science University (IACUC approval #IS00001801). All images were grossly segmented manually prior to machine segmentation to eliminate regions outside the cellular layer or region of interest (RoI), and then batch-loaded (using the EBImage package in R [11]).

The R segmentation procedure entails calling an image for processing, applying algorithms, and summarizing the results. Each called-up image was converted to a rasterlayer using the ‘spatstat’ and ‘raster’ packages in R [12]. A rasterlayer is a single layer of raster data, in which each pixel is represented in a raster coordinate reference system, with a unique set of coordinates. The coordinate grid in the raster data structure provides the basis for map algebra operations. The coordinate grid indexes pixel values by location, meeting the prerequisite in map algebra for standardized access to corresponding pixels in one or more images (rasterlayers), and enabling operations between rasterlayers. Newly-calculated pixel values are stored in a corresponding coordinate grid, allowing for selection of raw pixel values from the original image based on the calculated pixel value in the masking image. This facility for storage and exchange is the foundation for extracting local and global features. Pixel values in the masking image are normally the basis for selection from the original image.

### Map algebra

Map algebra, used for communicating between the original image and a masking image with identical coordinates, enables local, global, focal, or zonal transformations (within the R raster package) of the original image [12]. In this study, we used the focal function, which by default replaces pixels with a neighborhood mean.

**Fig 2:**
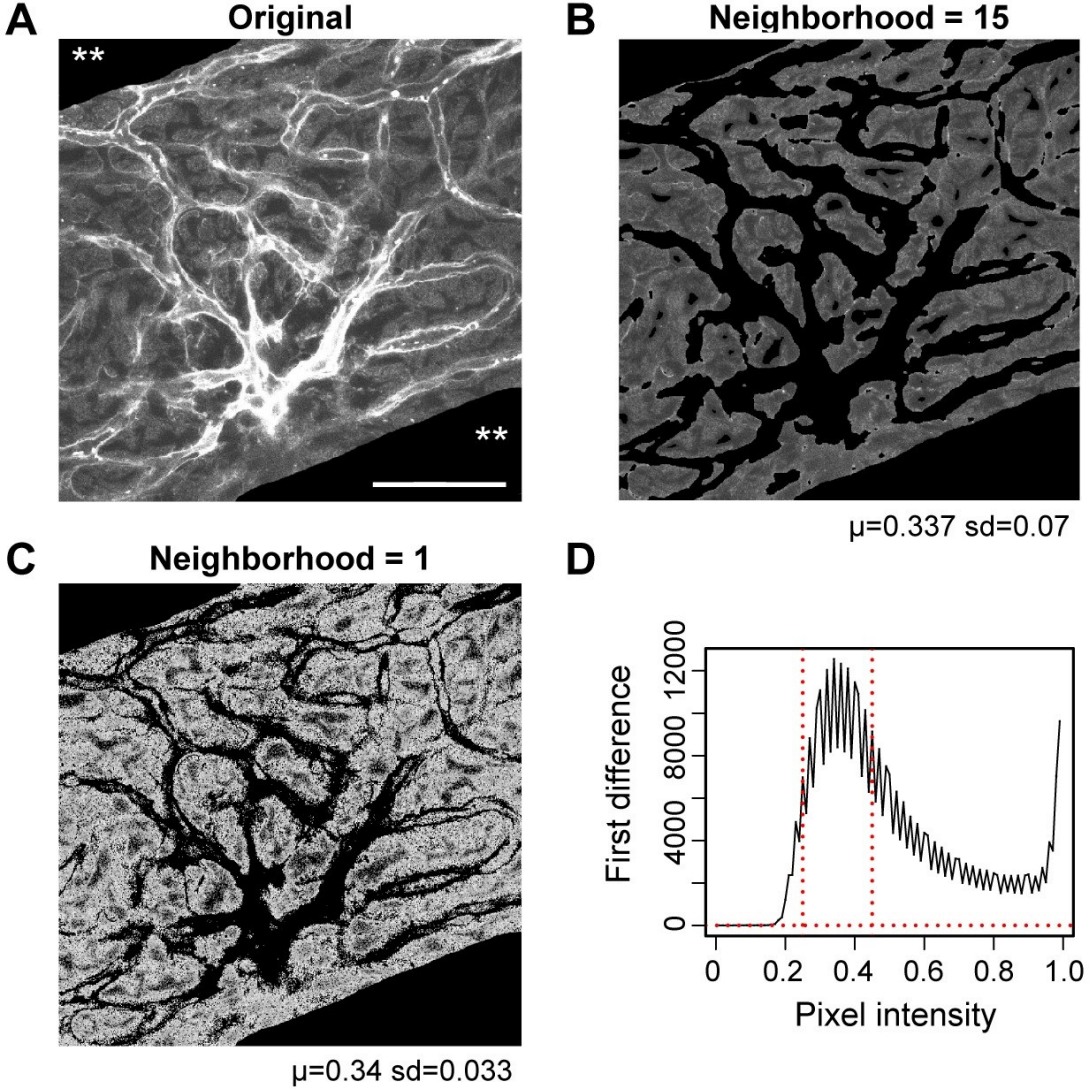
Map algebra-based mean smoothing. **(A)** A raw confocal image of an intermediate cell layer, with gross segmentation to remove extraneous cell layers (**). **(B)** The original image (in A) was focal function mean smoothed with a 15x15 neighborhood. In the masking image, pixels were replaced by the mean of their neighborhood, and then segmented, in this case, with a percentile algorithm. This image resembles a manually segmented image using digital erasers. Note the border around the image, due to the indeterminacy of the pixel calculation within 15 pixels of the edge of the original image (mean pixel intensity, μ = 0.337, sd, standard deviation). **(C)** This image is segmented without mean smoothing (smoothing neighborhood = 1, μ = 0.34). Although the image is visibly brighter than in (A) or (B), this is an artefact of R processing, and does not affect the underlying pixel values following segmentation. **(D)** Graphical representation of the first difference for the segmented image in (A). Note the noisiness. Scale bar in A = 50 *μ*m and also applies to (B,C).

Selecting pixels with a focal function-based mean filter effectively eliminates anomalous pixels (Fig 2 illustrates this in a percentile-segmented image). Since the discrepancy between the neighborhood mean and original pixel value is a measure of the conformance of the pixel with neighboring pixels, the mean filtering implicitly indicates the degree of local autocorrelation (local homogeneity). Pixel values not sufficiently in accord with neighboring pixels in the original rasterlayer are skipped as discrepant pixels when selecting from the original image rasterlayer. The effect of the mean filtering is to smooth articulation of the segmentation and reduce calculation time (shown indirectly in Fig 2). The reduction in calculation time is the result of a less noisy first or second difference curve to process–the noisiness is shown in Fig 2D. The apparent effect of mean smoothing over a larger neighborhood is segmenting with a wider brush (compare Fig 2B and 2C). The image segmented in conjunction with 15x15 neighborhood mean filtering displays with a higher standard deviation (due to stronger contrasts between consolidated segmented and unsegmented regions).

Lastly, we must mention the importance of the masking image from a programming point of view as a short-term memory ‘sketchpad’. This sketchpad enables interaction with pixel values in the original image. The masking image enables selecting values from the original image based on calculated values in the mask. This enables multi-level analysis, blending together different types of information, e.g., neighborhood mean, global or local quantiles, and local autocorrelation. With this multi-dimensional capability, different features of the image are logically conjoined to serve as a basis for selection and analysis.

Focal functions are also well suited for identifying other local features, since focal functions are by definition calculated from specified neighborhoods. The matrix used to calculate new pixel values in the focal function is a convolution kernel that can utilize other functions or be customized. Although we utilized the mean (default) filter and local Moran autocorrelation in the experiments described, reasonable algorithms can also be implemented incorporating a variety of other kernels, including the mode, maximum, or customized kernel.

### Integrated intensity-based methods

**Fig 3:**
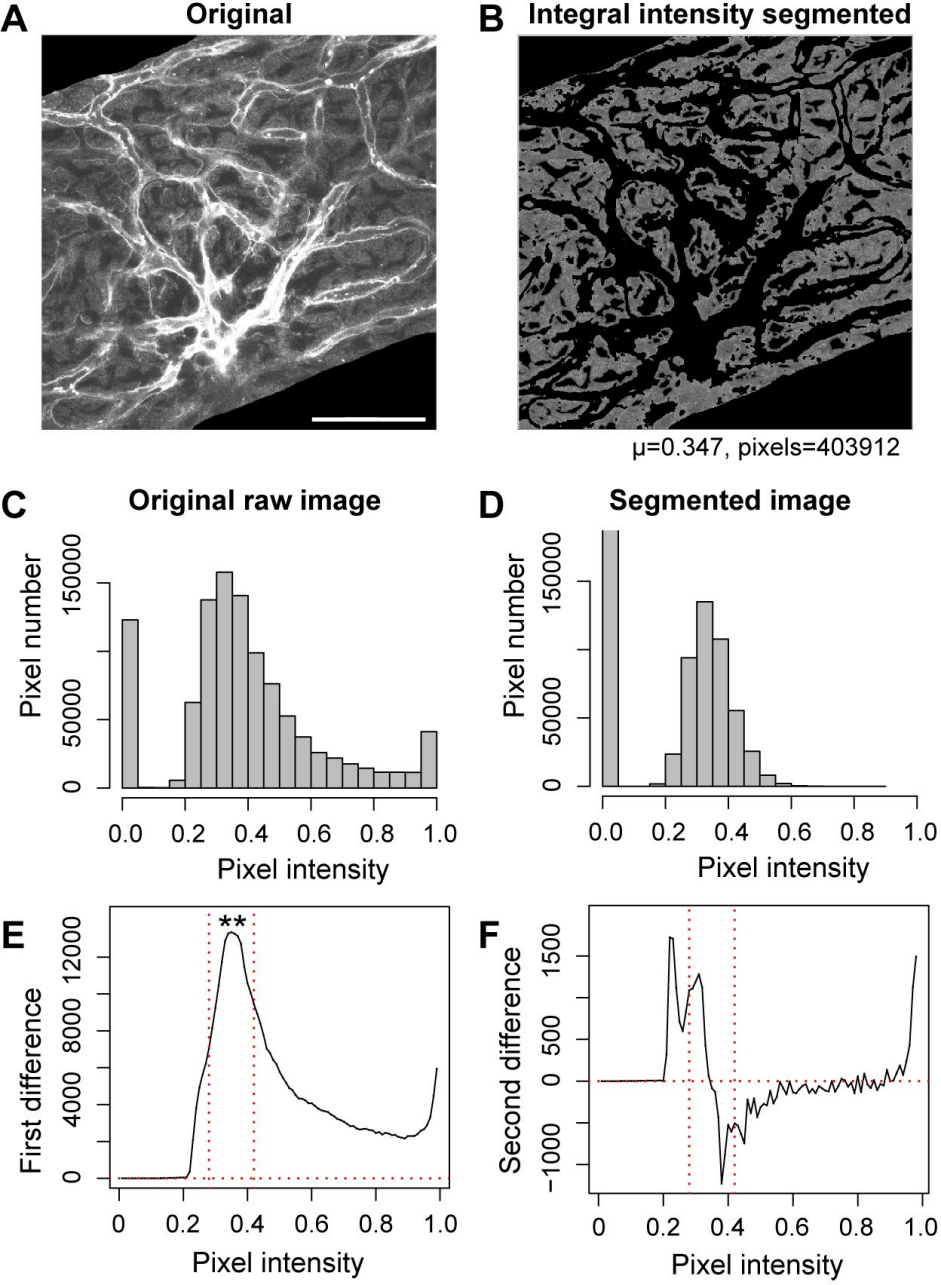
Integrated pixel intensity-based segmentation. **(A)** A raw confocal image of a gross-segmented intermediate cell layer. Scale bar = 50 *μ*m, and also applies to (B). **(B)** The image in (A) was mean smoothed with a 7x7 pixel neighborhood, and segmented using a first difference integrated intensity-based algorithm. **(C,D)** Histograms showing the distribution of pixel intensities in the raw and segmented images in (A) and (B). Note that the remaining non-zero pixels in the segmented image (B) approximate a normal distribution, unlike the skewed distribution in (A). **(E,F)** Difference curves drawn from integrated pixel intensity analysis of the original image. The optima (**) of the first difference (E) is shown with a fixed threshold interval set around the optima (between the vertical red dotted lines, in this example 0.07 to either side of the optima on the normalized [0,1] scale), and represent the pixels retained by segmentation for subsequent statistical analyses. **(F)** The second difference curve is shown for comparison. Second difference segmentation involves more boundary condition complexity, yet the results are similar. The second difference downward root crossing is identical with the first difference optimum. For (C-F), the *x*-axis is normalized, and for (C) and (D), the *y*-axis refers to the number of pixels in the original or segmented image.

The foundation of integrated intensity-based methods is watershed (gradient-based) thresholding (Fig 3). We identified mathematical markers to define the predominant distribution of interest (in this case, cytoplasmic areas) in our images. Generally, as described in more detail below, an optimization is performed on the vector of integrated pixel intensity to obtain upper and lower thresholds. These were typically applied to the focal mean smoothed masking image, and the pixel distribution of the image was qualitatively changed. A histogram of the frequency of individual pixel intensities in the raw image was typically a skewed distribution (Fig 3C), while the segmented image more closely approximated a normal distribution (Fig 3D).

Different regions in a confocal image show different rates at which pixel intensity is changing (Fig 3E and 3F). These are useful for segmentation since the rate of change in the integrated pixel intensity maps to changes in morphology in the image (Fig 3B). The integrated pixel intensity was calculated as a cumulative sum for each pixel value in a [0,1] range, the product of the interval frequency and pixel intensity. The integrated intensity is a vector of 100 values. The rate of change in the integrated intensity (first difference) is a vector of 99 values obtained from differencing subsequent elements of the integrated intensity vector, and likewise the second difference is a vector of 98 values obtained from differencing subsequent elements of the first difference. Flow charts for a sequence of steps in integrity-based segmentation (first and second difference) are shown in S1 and S2 Figs. Graphs of the first and second difference are shown in Fig 3E and 3F. The plot of the first difference demonstrates a conspicuous optimum (Fig 3E). Likewise, the plot of the second difference has a distinct horizontal ‘S’-curve, with “root crossings” where the curve crosses y = 0 (Fig 3F). The optimum and root crossings are readily identifiable reference points in pixel intensity space. Both optimum and roots are readily determined by appropriately filtering the first and second difference vectors. For example, the optimum of the first difference is determined by locating the index of the vector (pixel intensity on the *x*-axis) for which the first difference vector reaches a maximum (in frequency) before decreasing in value. Similar calculations can be made using the second difference vector, as the optimum on the first difference vector corresponds with the downward root crossing in the second difference vector. However, caveats apply when using the second difference vector, as the curve for the second difference vector is noisier than the first difference vector. For example, the noisiness of the ascending root crossing makes determination of the asymptote of the second difference curve difficult (Fig 3F), and, if the asymptote is to be utilized in the algorithm, an arbitrary choice must be made how to terminate it. Root crossings in the second difference vector, other than the primary downward root crossing, are at best only *initial* references for estimating the low and high segmentation thresholds. In lieu of more sophisticated second difference methods to set these thresholds, these thresholds are normally set in first difference methods as a fixed unit of image intensity to either side of the optimum (Fig 3E). Alternatively, determination of the thresholds can be based on the degree of curve inflection. In practice, given the problematic noisiness of second difference methods, and the ease with which the simple optimum of the first difference is identified, the second difference methods are now largely avoided.

In general, the objective in integrated intensity approaches is to map morphology relative to mathematically salient points and to use the mathematical handles as a means for separating regions. The map algebra used in conjunction with integrated intensity-based methods is embedded in the method (since selection from the original image is normally based on values in the masking image). Use of map algebra for smoothing the original image, or for more sophisticated multi-dimensional combining of different approaches, is secondary to its primary use in providing common pixel coordinates for selection of pixels for intensity analysis.

### Percentile-based methods

**Fig 4:**
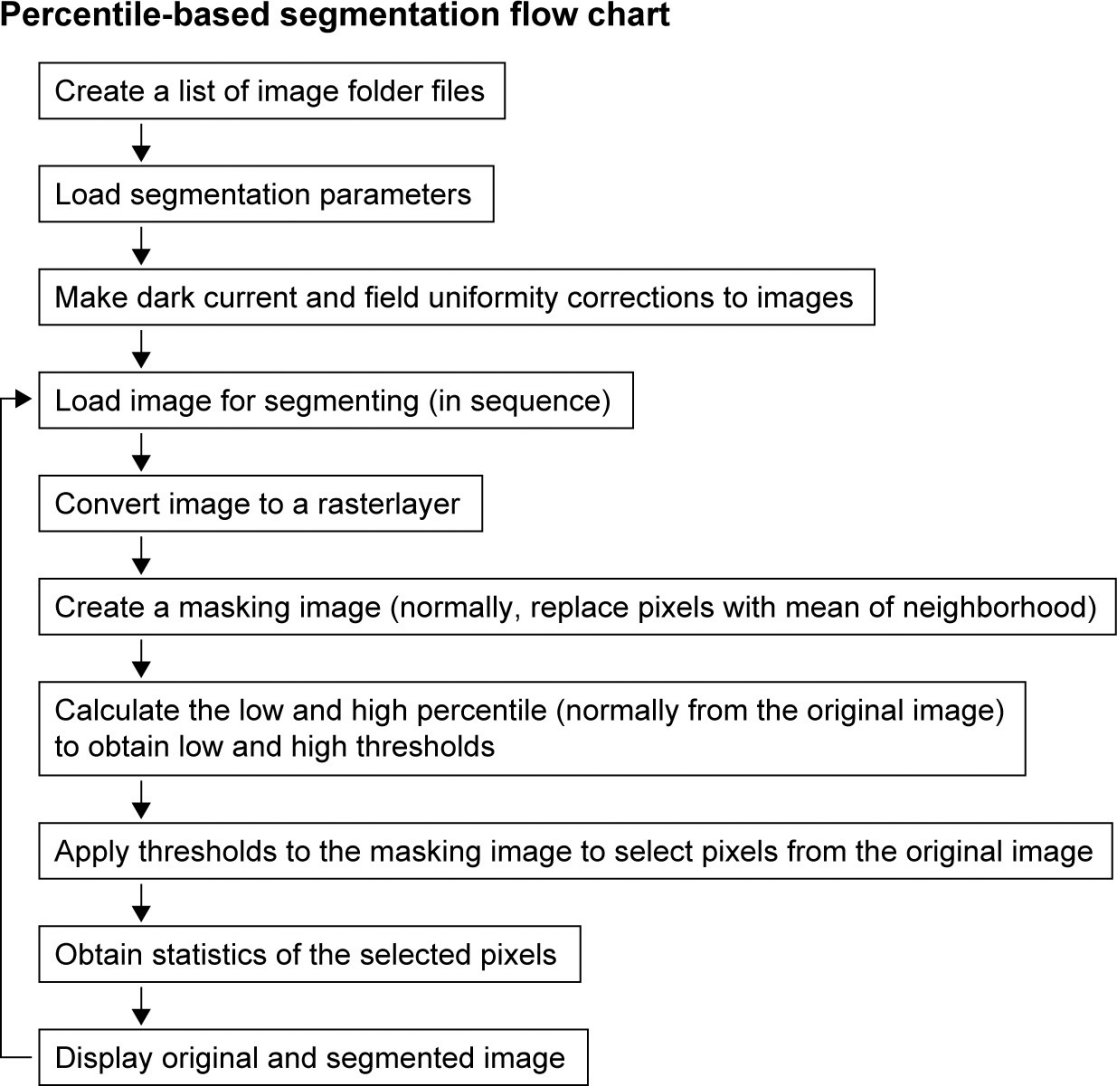
Flow chart for percentile-based segmentation. Sequence of steps to accomplish percentile-based segmentation.

**Fig 5:**
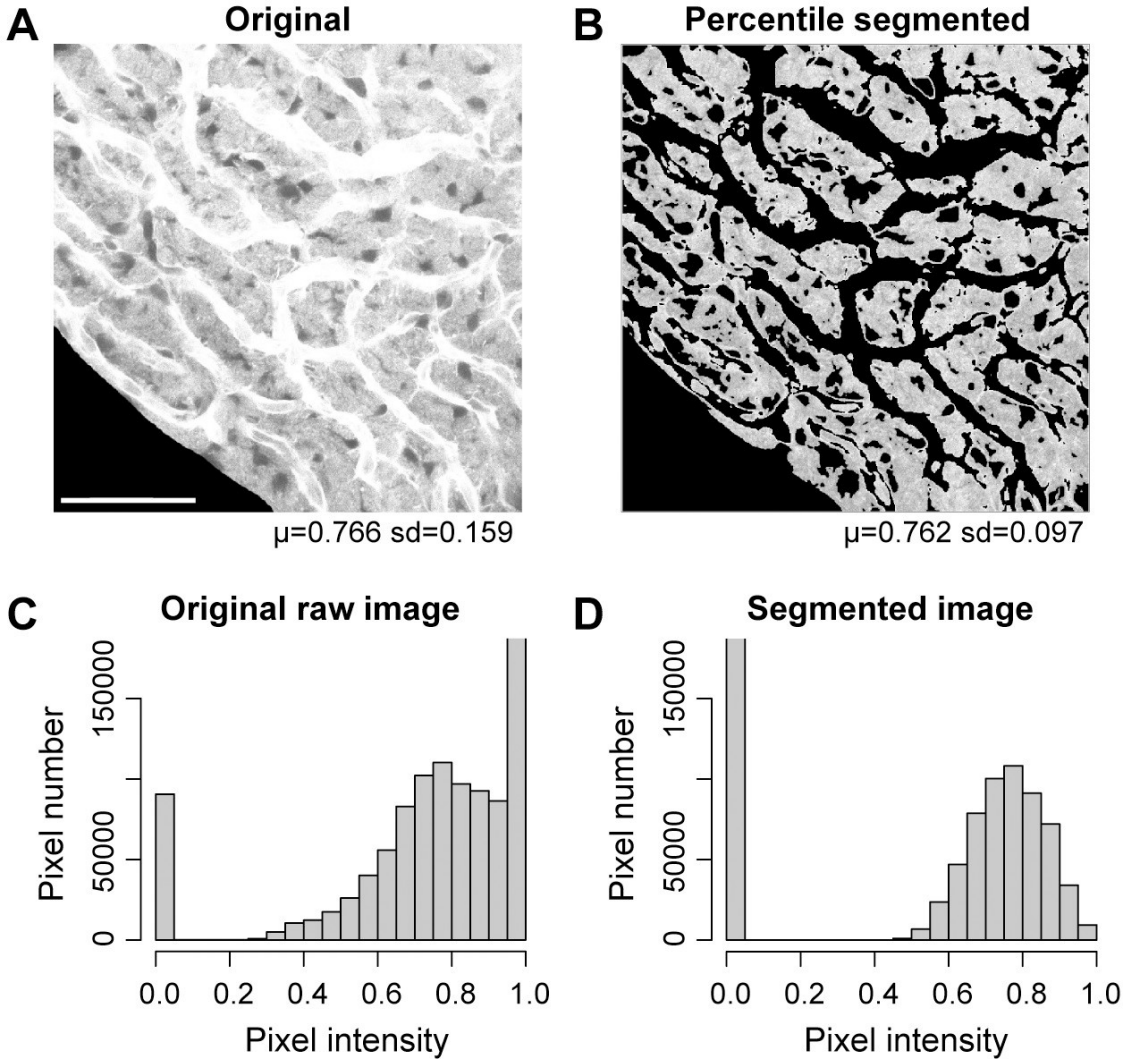
Percentile-based segmentation. **(A, B)** The original and percentile-segmented image obtained when setting the upper threshold to the 70^th^ percentile and the lower threshold to the 20^th^ percentile of the original image, and then selecting pixels from a mean smoothed image (7x7 neighborhood) at these threshold values. Note the decrease in the variance (s.d.) in the segmented image. Scale bar in (A) = 50 *μ*m, and also applies to (B). **(C, D)** Histograms of the raw and percentile-segmented images shown for comparison purposes.

**Fig 6:**
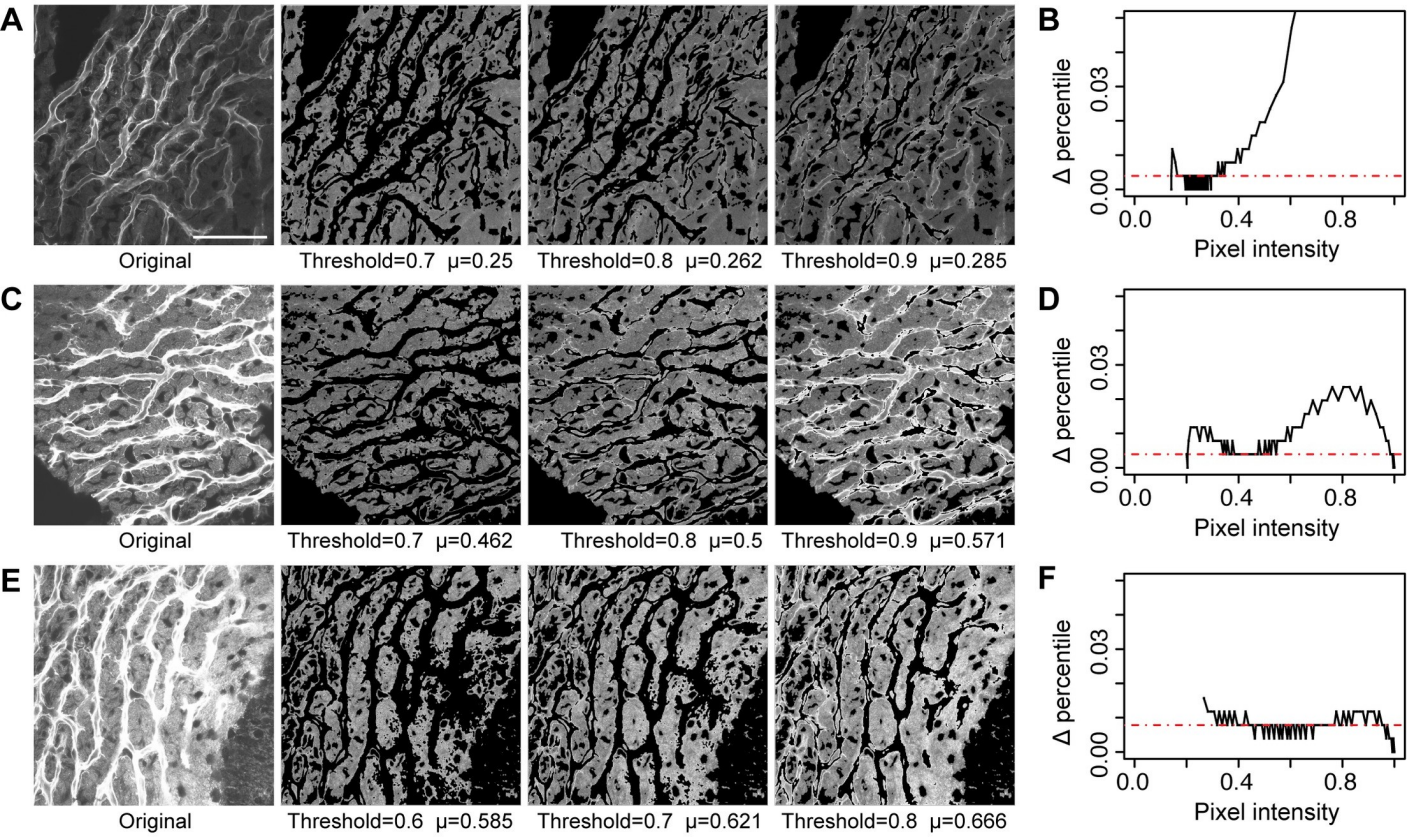
Sensitivity of threshold to percentile segmentation. All images were percentile-segmented. ‘Upper threshold’ is given as a percentile, expressed as a decimal between 0–1 below each image. **(A)** A dim image segmented at a range of upper thresholds. Scale bar = 50 *μ*m, and also applies to (C) and (E) as well. The accompanying graph **(B)** displays the first difference of the percentile as a function of pixel intensity in the original image. For this image the change in the percentile is flat over a range of pixel intensities from 0.15 to 0.3 (corresponding to the 20^th^ and the 70^th^– 90^th^ percentile). **(C)** A medium bright image segmented at a range of upper thresholds. **(D)** The first difference of the percentile is flat for a range of pixel intensities from 0.3 to 0.55 (corresponding to the 20^th^ and the 70^th^– 90^th^ percentile). **(E)** A bright image segmented at a range of upper thresholds. **(F)** The first difference of the percentile is flat for a range of pixel intensities from 0.45 to 0.75 (corresponding to the 20^th^ and the 60^th^ - 80^th^ percentile). In all cases, the best segmentation upper threshold is the point at which the basin of the percentile first difference inflects upward. The dotted red line in **(B,D,F)** is the mode of the percentile first difference. Note the divergence from the mode for these 3 images is in the vicinity of percentiles between 20^th^ and 70^th^ percentile. In addition, thresholds expressed as a percentile are relatively constant, while thresholds expressed as pixel intensity vary widely.

A flow chart of the sequence of steps for machine-based percentile-based segmentation is shown in Fig 4. Percentile-based segmentation (Fig 5) is based on the percentile curve of the region of interest (RoI; i.e., the grossly segmented images). Compared with the complexities of integral intensity methods, the percentile method is simple. The percentile indicates the pixel intensity for which the percentage of pixels in the image is equal to, or less than, the referenced percentile. Effectively, the image is divided into 100 bins, and each bin (in this case equivalent to percentile) represents the maximum pixel intensity in each group of 10486 pixels (1048576 pixels per image divided by 100). In the Fig 5 example, the original image was mean smoothed over a 7x7 neighborhood to give a masking image and selection from the mask on the original image included the corresponding pixel intensities from 20^th^ and 70^th^ percentile obtained from the original image. Percentile methods effect a transformation of the image data into a percentile framework. The percentile cutoffs become relatively invariant pointers to the pixel intensity thresholds (Fig 6). Setting the thresholds at the 20^th^ and 70^th^ percentiles is relatively stable relative to the wide range of thresholds in terms of pixel intensity. As is the case with integral intensity methods, percentile segmentation reshapes the pixel intensity distribution. The histogram of the original image (Fig 5C) is skewed, while the histogram of the percentile segmented image (excepting the zeroed pixels) approximates a normal distribution (Fig 5D). Although the percentiles are calculated for the original, raw image or RoI (after gross segmentation), the percentile-based thresholds are typically applied to a mean smoothed image. An R package to perform percentile segmentation is being prepared for inclusion in BioConductor (https://www.bioconductor.org/).

### Local autocorrelation-based methods

**Fig 7:**
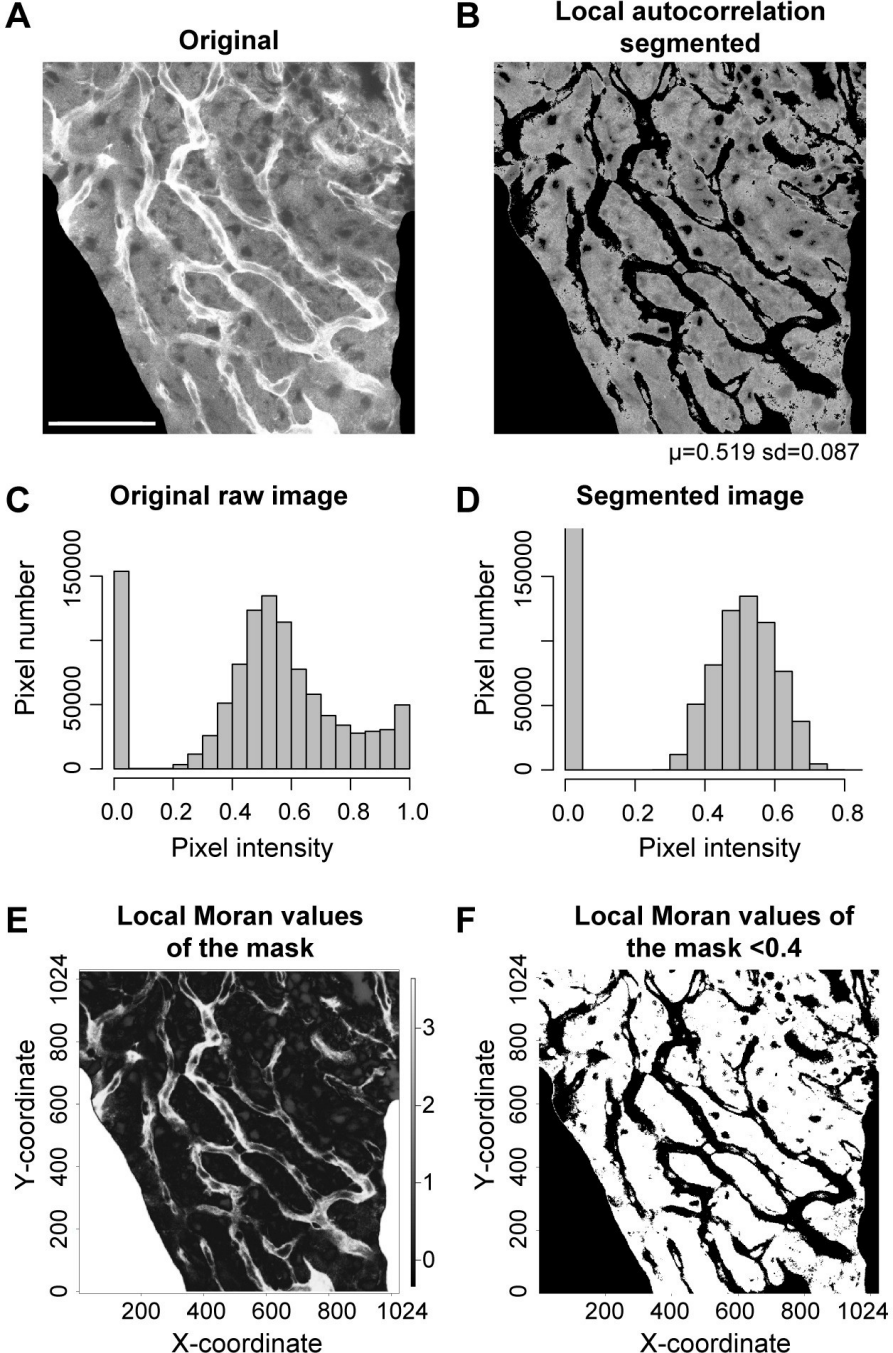
Moran local autocorrelation-based segmentation. **(A,B)** Original and local autocorrelation segmented image. The threshold on the Moran parameter was set at 0.4. Since both low and high pixel values tend to be local autocorrelated, the interval for accepting pixels extends from the lowest Moran value to the cutoff at 0.4. Scale bar in (A) = 50 *μ*m, and also applies to (B, E and F). **(C,D)** Histograms of the raw and local autocorrelation segmented images. **(E)** The MoranLocal function is used to create a focal masking image. Shown are the local Moran values of the masking image ranging between -0.532 and 3.64. Note the similar autocorrelation of the removed image area (all zeros) and the saturated staining of vessels. **(F)** A logical function defining pixels with MoranLocal less than 0.4 turns the masking focal image (E) into 0s and 1s. This logical mask is used to select corresponding pixels from the original image for analysis.

**Fig 8:**
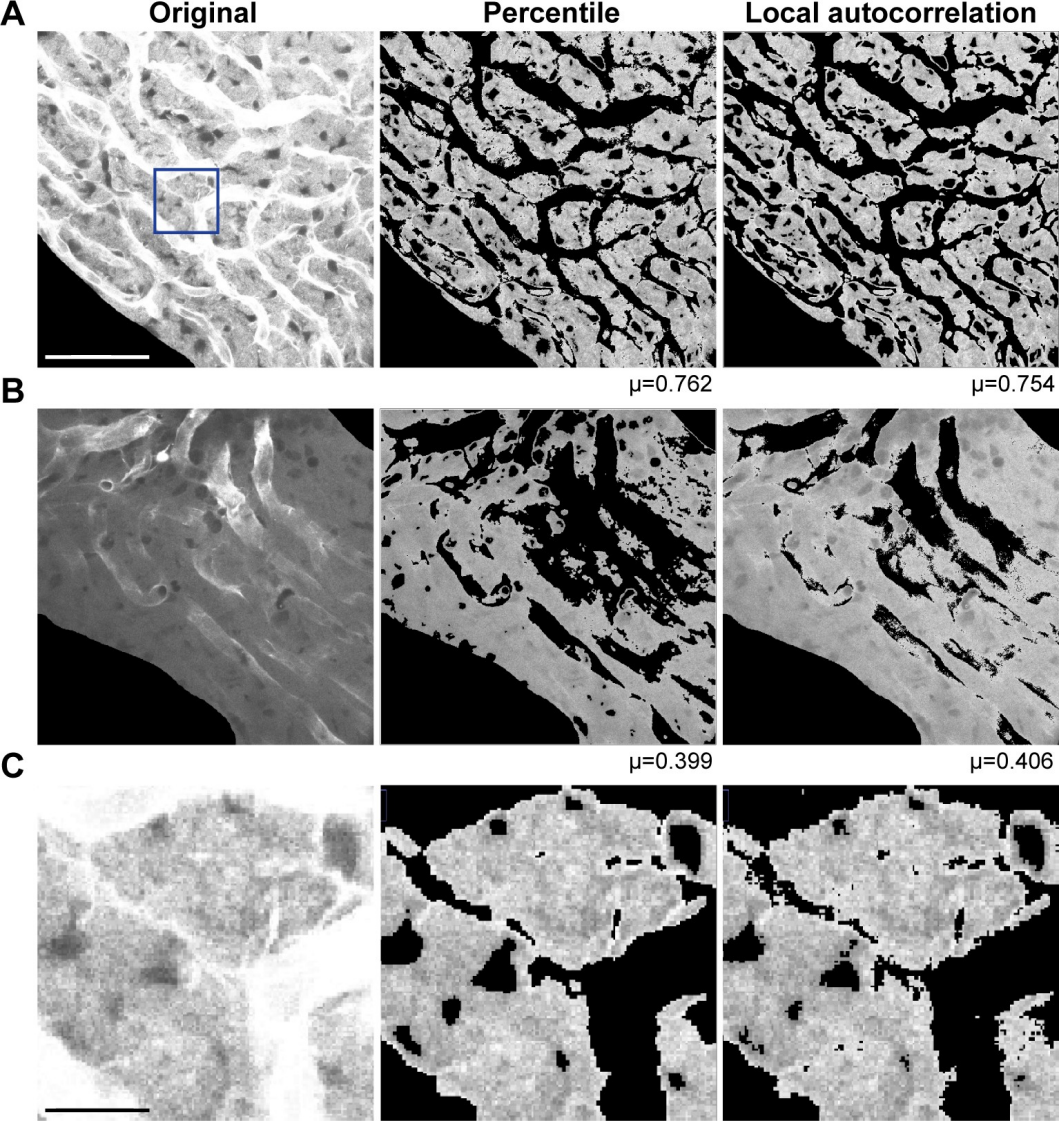
Percentile- and local autocorrelation-based segmentation methods are comparable. Percentile segmentation with thresholds of 0.2 and 0.7 (20^th^ and 70^th^ percentile) and a 7x7 focal neighborhood is compared to local autocorrelation segmentation with a Moran cutoff value of 0.4 and a 7x7 neighborhood. **(A)** Percentile and local autocorrelation segmentation of a bright image. Scale bar = 50 *μ*m, and also applies to (B). **(B)** Percentile and local autocorrelation segmentation of a medium bright image. **(C)** Zoomed comparison of percentile and local autocorrelation segmentation of the box in the original image in (A). Even without adjustment of parameters, segmentation appears almost identical. The apparent shift in brightness is a programming artefact. Scale bar = 10 *μ*m.

Local autocorrelation is a measure of localized homogeneity. For a given neighborhood, the local Moran autocorrelation (Fig 7, [12]) is a measure of the similarity of its pixel values [13]. For example, a homogeneous area would give high local autocorrelation. We define localized similarity for every pixel neighborhood of the original gross segmented image and create a masking image of the local autocorrelation (Fig 7E and 7F). Local autocorrelation, as distinct from global autocorrelation, is the measure of the local homogeneity in the defined locale surrounding each pixel. Local autocorrelation is implemented using the map algebra focal function with ‘MoranLocal’ (in the raster package of R) as the convolution kernel [13]. Calculation of local Moran affords some simplification over other methods of segmentation since both background and oversaturated regions are relatively high in autocorrelation (a benefit obtained at the expense of increased time needed for computational processing). Segmentation using local autocorrelation methods was quite comparable with integrated intensity or percentile methods (compare Figs 3B and 5B with Figs 7B and 8). Like other methods, local autocorrelation-based segmentation resulted in an approximately normally distributed segmented image (Fig 7D). A flow chart of a sequence of steps in local autocorrelation-based segmentation is shown in S3 Fig.

### Supervision

The common denominator in effective machine-based automated segmentation is supervision. Supervision involves initial checking of individual image results and robustness, and then fine-tuning algorithms until results approximate those expected under manual segmentation. Robustness in this case generally means smooth transitions in results where smooth transitions are expected (manually segmented results are the comparative basis for evaluation). However, the evaluation is not purely subjective, as there are also objective criteria; e.g., the algorithm should give results without wild gyrations to small perturbations in input. Fine-tuning can include substantive revision of the algorithm or choice of a different algorithm and adjustment of parameters. Supervision was performed before batch processing, however even after processing large numbers of images, the results were checked for anomalies (results not in accord with visual expectations) prior to statistical analysis.

### Statistical analysis

The primary objective of this study was to develop methods for automating segmentation and statistical analysis of image intensities in confocal images. This objective is only satisfied if results obtained from automated analysis mirror results obtained from manual segmentation. To meet this requirement, we expect robustness, consistency, and comparable results when statistical tests are made on the manual and machine segmented images. Tests included both examination and formal statistical tests. We examined images to verify the absence of systematic bias at the pixel level, and then tested pairwise sets of manual- and machine-segmented images. We examined the consistency of machine segmentation across the *z*-stack. Gradual changes in the segmentation, as opposed to abrupt changes, are preferred. We tested the robustness of the segmentation to arithmetic translation of the image. We verified whether the trends of manual- and machine-segmented image sets were consistently biased. Finally, and most importantly, we affirmed whether differences found between experimental conditions under manual segmentation were preserved under machine segmentation.

## Results

Initially, we describe outcomes for integrated intensity-based methods involving first and second difference methods, as these are more intuitively understandable. Subsequently, we describe outcomes from percentile and local autocorrelation methods, culminating with evaluation and validation. Additional details and the scripts can be found in the Supporting Information section (S1 Data). We discuss the relative efficacy of each approach in the Discussion section.

### Integrated intensity-based methods

The general heuristic was to map fluorescence intensity in a tractable manner to find mathematical handles correlating tissue morphology with distribution of pixel values in the confocal images. By calculating optima, slopes, and root crossings, we obtained a qualitative sense of where pixel values were changing most rapidly in pixel intensity space. The changes in pixel intensity ‘velocity’ (first difference) and ‘acceleration’ (second difference) correspond to changes in morphology in the confocal image. As we step through the integrated pixel intensity we find regions where the rate of change in pixel intensity increases and decreases, and similarly for increases and decreases in the difference of this rate. For example, cell membranes in images are frequently sites of abrupt change in pixel intensity, and these pixels can readily be identified by first difference methods as an uptick in the integrated pixel intensity. In second difference methods, the cell membranes display as a transition from an increasing to a decreasing rate of change. Correspondingly, the moving average of the integrated pixel intensity undergoes subtle changes as we step through pixel intensity space. Thus, both the optimum for the first difference and root crossings for the second difference are salient reference points for segmenting out morphological regions of interest. Choice between first and second difference method is largely a choice which provides clearest signal.

#### First difference method

The first difference of the integrated intensity vector was filtered to identify the pixel intensity at which the first difference curve reached its peak value (the optimum) and began decreasing in numerical value (Fig 3E). The method involves finding the pixel intensity for which the pixel intensity to either side has lower frequency. The optimum is also the value of the descending root crossing for the second difference, i.e., the pixel intensity at which the second difference curve crosses from positive to negative value (Fig 3F). By focusing on the optimum of the first difference, we implicitly assume that the RoI can be identified relative to an interval where the integrated intensity undergoes a transition. We focus on these markers of transition, and reject regions of the first difference curve that represent background (or dark current), or overly bright (or saturated) pixels where the first difference curve extends off the scale. After determining the optimum of the first difference, we made some assumptions about the distribution of pixel intensities, e.g., we assumed the frequency of pixel intensity around the optimum is symmetrical, and set the RoI cut-off thresholds at a fixed interval to either side of the optimum. Alternatively, the intervals could also be set to either side of the optimum determined by inflections in the first difference curve. These different alternatives were set as choices in the algorithm. We obtained and applied these thresholds to a mean filtered image.

Empirical validation is a crucial part of algorithm design. We empirically optimized the first difference method by comparing manual segmentation results with our reading of the first difference curve. This revealed the importance of the optimum of the first difference in demarcating regions of morphological interest. The pixel value corresponding to the optimum of the first difference was a good estimate for the mean pixel intensity of cytoplasmic fluorescence. The fitness of this segmentation is demonstrated in Fig 3C, where the skewed histogram of pixel values in the original image contrasts the normal distribution after segmentation (Fig 3D). Successful estimation of thresholds from the integrated intensity vector for the first and second difference underscores the importance of supervision in the development stage. Supervision at this stage involves tweaking the calculations based on the first (or second) difference of the integrated intensity vector to ensure reliable identification of salient morphological features in the image (see below for further discussion).

#### Second difference method

Second difference methods are similar to first difference methods, however second difference algorithms are more complex. In second difference methods, we step through pixel threshold values, and note the rate of change in the integrated pixel intensity value varies. The second difference reflects a moving average of changes in the overall mean as pixel intensity increases to values characteristic of the cytoplasmic RoI. There are similarities with first difference methods. For example, the downward root crossing corresponds to the first difference optimum (Fig 3E); yet this method produces additional information. The ascending root crossing (subsequent to downward root crossing) was found to be a rough estimate of the upper segmentation threshold, and while the noisy asymptote is a good starting point for identifying the upper threshold of the cytoplasmic distribution (Fig 3F). Refinement of threshold determination can employ different strategies, including determining inflection points where the second difference curve has significant changes in slope. The noisiness of the second difference vector can be problematic, and different strategies can be employed to minimize noise and determine realistic inflection points. One strategy is to enlarge the neighborhood of mean filtering; this smooths the noisiness of the curve and makes determination more tractable. Asymptotic sequences make it difficult to define a clear root. Strategies were employed to terminate asymptotic sequences of the upper threshold to eliminate background and over-saturated regions from inclusion within the pixel selection mask thresholds. After paring away unrealistic values and deciding on a heuristic for determining thresholds, supervision and visual examination were employed to refine the algorithm. Examination of the segmented images is the final arbiter for determining the usefulness of the adjustments.

### Percentile-based methods

In percentile-based segmentation, pixel intensity thresholds are set at fixed percentiles, e.g., the lower threshold at the 20^th^ percentile, and the upper threshold at the 70^th^ percentile (Fig 5). The percentile indicates the percentage of pixels in the image equal to, or less than, the referenced pixel intensity. The profile of the pixel intensity by percentile is a measure of the ‘nonlinearity’ in the pixel intensity distribution: percentiles expressed in the [0,1] range do not match pixel intensities expressed in the [0,1] range. The discrepancies are indicators. Deviations from a linear profile are indicative of an accelerating or decelerating rate of change in the cumulative pixel intensity. In other words, there is bunching of the pixel intensity values in pixel intensity space.

Percentile-based methods are uncannily simple, at least on the surface. The question is why percentile methods are effective. As noted above, percentile subsets do not map proportionally to pixel intensity. The differential rate of growth in the percentile with respect to pixel intensity is a measure of this nonlinearity. The rate of change can be graphed relative to pixel intensity (every percentile maps to a pixel intensity, Fig 6B, 6D and 6F). The graph of the rate at which the percentile changes as a function of pixel intensity is a concave “U”-shaped curve with a prominent central plateau. The change in percentile is reflective of the number of pixels at a specific pixel intensity. Since change in percentile, “Δ percentile” in Fig 6, corresponds to number of pixels, it also reflects homogeneity. The central plateau, marked relative to the mode of the images (blue dashed line), represents the region of pixel intensity space which is relatively heterogeneous. In the plateau region of the “U”-shaped curve, increments in the pixel intensity give marginal increments in the corresponding rate of change in the percentile. Conversely, the upward ticks at the ends of the “U”-shaped curve are ‘thin’ in the sense that small differences in pixel intensity correspond to large rates of change in the percentile (greater numbers of pixels at that intensity). The upward tick in the percentile rate is indicative the pixel intensities in these ‘thin’ regions are autocorrelated. Estimated thresholds for segmentation of the cell cytoplasm are the pixel intensities corresponding to the end points of the plateau. If the two ascending extrema of the percentile first difference are clipped off, the plateau region represents the cytoplasmic region of interest, and the range of the plateau is a reasonable benchmark for setting thresholds. The method identified where pixel intensities are compressed together in percentile space (‘thin’ ends) and where they are less compressed (plateau). Thus, in the percentile method, we can determine the lower and upper pixel intensities for thresholding based on the degree that pixel intensities are bunched together. Importantly, while the range for determining reasonable thresholds using pixel intensity is wide across different types of images, the range for reasonable thresholds by determined by percentile-based methods is relatively stable. In our images, percentiles set at 20% and 70% (20% of pixel values are ≤20^th^ percentile and 30% are >70^th^ percentile) work well for isolating parenchymal areas, and were essentially stable over the entire extent of image brightness (Fig 6).

As with integrated intensity-based methods, segmentation cut-off thresholds were normally applied to a mean neighborhood filtered masking image, although thresholds can also be applied without the mean filtering. Some trial and error may be involved in setting appropriate percentiles for images other than the intermediate cells the method was developed for, but once identified, this method was surprisingly robust across images, image stacks, and images from different experimental groups.

### Local autocorrelation-based methods

Local autocorrelation is a common underlying theme in calculus- and percentile-based methods. The heart of these methods is taking the measure of a similarity, i.e., distinguishing the local similarity of one morphological feature from another, and demarcating it. In integrated intensity-based methods, this similarity is determined from a rate of change or moving average of the integrated pixel intensity. These differential rates of change can indicate homogeneous and heterogeneous intervals in pixel intensity space. For example, growth in a moving average shows identifiable pattern over ranges of homogenous and heterogeneous pixel intervals (Fig 3E and 3F). We have shown how this differential statistical feature can be used to set morphological features apart. A similar opportunity presents in percentile-based methods. The key assumption is that morphological regions map to differential pixel frequency. In percentile-based methods, we filter out local autocorrelated regions, as these tend to be concentrated in compact intervals at low and high pixel values, leaving regions of more heterogeneous pixel intensity. The nonlinearity in pixel frequency facilitates the segmentation into morphologically similar ‘basins’, partly because the local autocorrelated regions tend to be relatively ‘thin’. Thus, this simple heuristic is surprisingly effective for separating out the locally autocorrelated background and stained vessels from the parenchymal areas (Fig 7).

But assessment of local autocorrelation can also be used directly as a criterion for segmentation. A single value of local Moran autocorrelation distinguishes the high autocorrelation of background and membranes from the lower autocorrelation of cytoplasm (Fig 7F). Segmentation by percentile- and local autocorrelation-based methods were essentially indistinguishable (Fig 8). As in integral intensity and percentile methods, the histogram of the segmented result was approximately a normal distribution (Fig 7D). Local Moran values do not fall in the [0,1] interval, ranging from negative to positive values, and, since the window which properly discriminates it is quite narrow, trial and error is required to find a value that distinguishes between lower Moran value cytoplasm and higher Moran value background and membranes. With some supervision, we determined a Moran coefficient of 0.4 is reasonable for most purposes.

### Supervision and validation

We have shown the utility of supervision for tuning several segmentation approaches to identify when changes in pixel intensity correlate with morphological RoI. Visual examination was the supervision standard. These included segmentation techniques based on first and second differences of the integrated intensity, percentiles, and local autocorrelation, each optimized by trial and error against visual examination of results. To evaluate the robustness of these algorithms, we verified them on images the algorithm had not been trained, and also compared our machine data with manually-segmented images. These tests were applied to both individual segmented images and image sets. Tests included between visual examination and statistical, with the most robust statistical tests done on percentile-segmented images.

**Fig 9:**
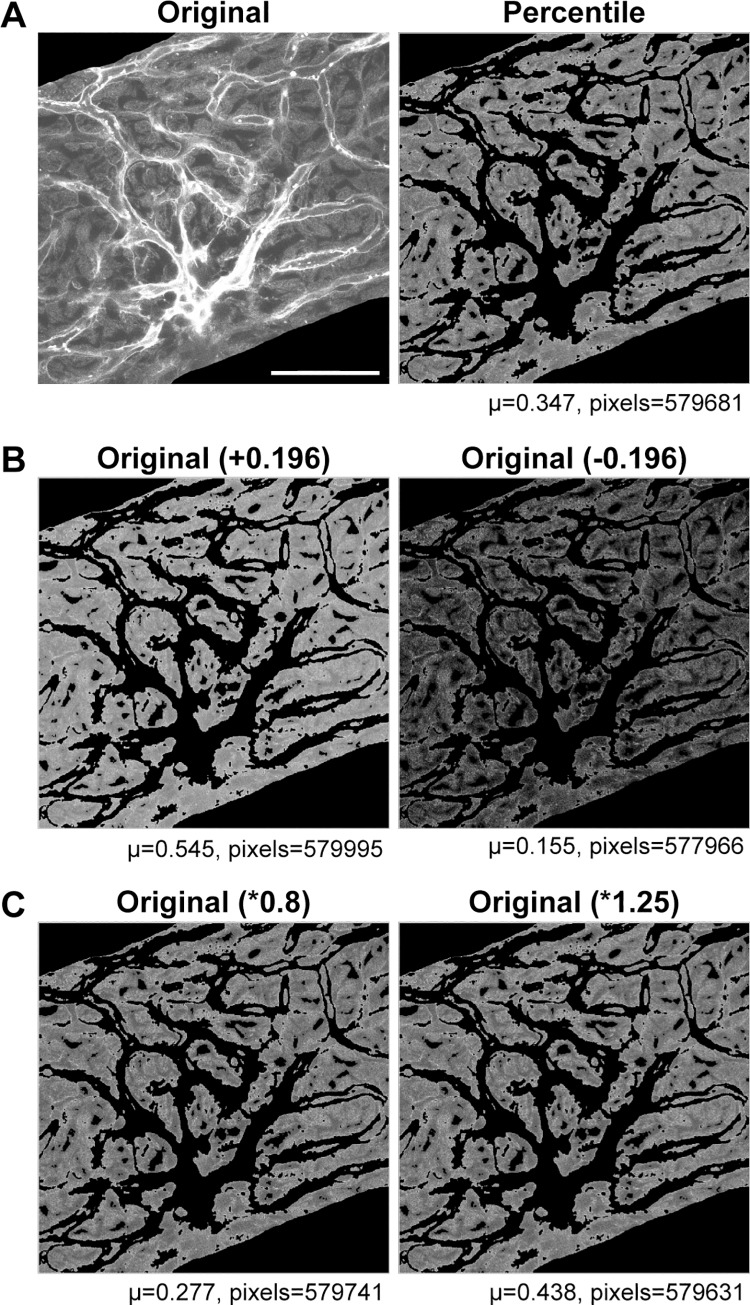
Robustness of percentile segmentation to mathematical transformation. The segmentation result should be robust, including robustness to pixel addition and multiplication. The means of images after addition and multiplication should accurately reflect the pixel transformation. **(A)** Original and percentile-segmented image (7x7 focal neighborhood). Scale bar = 50 *μ*m applies to all panels. **(B)** 0.196 [range 0,1] is added or subtracted to each pixel in the original. The segmented mean is within 1.5% of the expected mean. **(C)** Original image pixels are multiplied by a factor of 0.8 or 1.25. The segmented mean remained within 1.5% of the expected mean for the mathematical functions in both (B) and (C).

**Fig 10:**
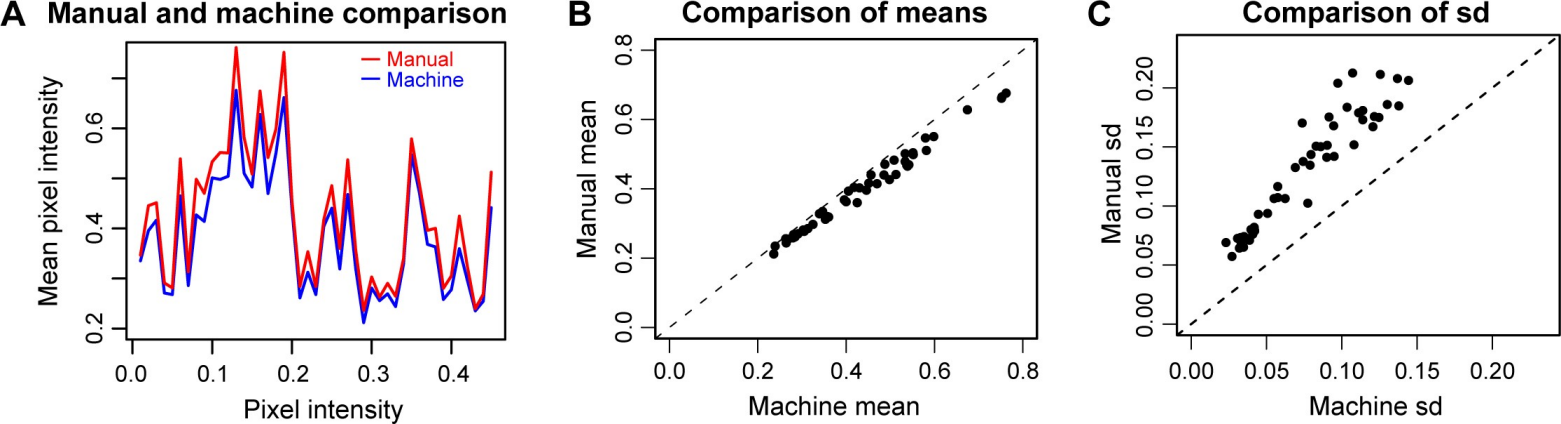
Comparison of manual and machine segmentation. **(A)** Manual segmentation (red) showed consistently higher mean pixel intensity than machine segmentation (blue) for different individual images. Cosine similarity ranging [–1,1], with perfect overlap equal to 1, is the inner product of the 2 non-zero vectors which represents the cosine of the angle between the two vectors. Vectors are compared of the means from manual and machine segmentation. The cosine similarity of the manual and machine vectors is 0.99. **(B)** Plot showing the consistent trend for manual segmentation to have a slightly higher mean value for each image. This suggests limitations in the fineness of articulation when manually segmenting. **(C)** Manual segmented images display with significantly higher variance. ‘Semantic’ manual segmentation leaves in place more heterogeneity than ‘syntactic’ machine segmentation. The standard deviation of the means of the manually segmented images is 0.173. The standard deviation of the means of the machine-segmented images is 0.106.

Comparison included examination of enlarged sections of the image (Fig 8C). When the image is enlarged, the individual pixels included or excluded by segmentation are easily monitored, and systematic problems can be localized. Other tests included perturbation tests, checking the robustness of the segmentation in image stacks, verifying similar statistical results, and ascertaining parallel trends in manual- and machine-segmented images. In perturbation tests, we made systematic, arithmetic changes to pixel values and compared the robustness of the method to the changes (Fig 9). In checking the robustness of image stacks, we found percentile segmentation results were consistent with changes in the morphology, while integrated intensity-based segmentation tended to be more variable. In verifying the correspondence between manual and machine segmentation, we verified that data trends were parallel and consistently biased. Descriptive statistics show that corresponding pairs of manual- and machine-segmented images were significantly different (*p*<0.5, Student t-test, see S1 Data). Yet the cosine similarity (>0.99) show that the trend of the manual- and machine-segmented images are parallel with a slight offset, the machine-segmentation having lower means (~2%) and variance (Fig 10). In general, the manual segmented images tended to have higher variance (Fig 10C). This is intuitive, since manual segmentation is based on identification of morphological structure, rather than a function of pixel intensity. The greater standard deviation of manual segmentation was confirmed, albeit with caveats. The manual and machine results showed lower variance pairs tended to be similar in variance, while higher variance pairs tended to be more variable.

When outcomes of manual- and machine-segmentation across experimental conditions were examined (S4 Fig), significantly higher mean pixel intensities were observed both machine- and manually-segmented images of toxin-treated compared to saline-treated strial tissues from a strain of transgenic mice (see S1 Data). In wildtype (control) mice, only marginal differences are seen between data from machine- and manually segmented images of toxin-treated compared to saline-treated strial tissues (see S1 Data [folder for Fig 10] and S4 Fig). These data emphasize that data from machine-segmented images replicated that from manually-segmented images, and with lower variance (Fig 10C). Overall, the results obtained under manual and machine segmentation were comparable.

## Discussion

Microscopy data are not fully quantitative. The constraints on interpretation of microscopy data have ramifications for quantitative comparison of microscopy images between experimental and control groups. In Stevens’ hierarchy of measurements [14], pixel intensities in acquired confocal microscopy images are largely interval data. Interval data are more quantitative than simple nominal (named) or ordinal (ranked) data, but less quantitative than ratio-scaled measurements with an absolute zero reference. Only ratio-scaled measurements can be normalized or used in a calibration curve; thus, the common practice in confocal microscopy is not to attempt rigorous calibration of pixel intensities. A number of conditions frustrate calibration, and, in particular, ratios of pixel intensity are particularly problematic. Fluorophore emission, read as pixel intensity, does not scale linearly with laser excitation, nor do equal increments of laser power produce analogous equal increments of fluorophore emission. The ratios of mean pixel intensities are not a simple function. As such, equal normalized ratios do not necessarily imply an analogous parallel in relative fluorophore emission. Furthermore, variabilities in the fluorescence intensity within the sample can obscure primary data, e.g., intense fluorescence outside the plane of focus can elevate the levels of fluorescence within the focal plane. This obscuration can be minimized by increasing confocality (i.e., by decreasing iris diameter), yet this out-of-focus influence is not completely eliminated. Spatial averaging also obscures the intensity of distinct features at scales finer than the optical resolution. These caveats serve to emphasize that pixel intensities are only suggestive of relative levels of cellular uptake of fluorescently-tagged compounds, not the definitive measure. Pixel intensity must be tallied with these limitations in mind, and thus, for the purpose of comparison, images of different samples from the same and comparison groups should be obtained under identical conditions as much as possible.

### Manual versus machine segmentation

Manual segmentation might be described as ‘semantic’ (‘instantaneous’ recognition of a pattern by humans), while machine segmentation, by contrast, is ‘syntactic’ (the algorithm proceeds in sequence and time). Although it could be argued that all semantic processes are essentially syntactic processes, on a phenomenological level the two are quite different. Human recognition has the quality of immediate synchronicity, while algorithmic ‘recognition’ is logical and time-dependent. The difference in processing is reflected in results and errors. On a phenomenological level, manual segmentation in our images tend to err on the side of too much variance, while machine segmentation tends to error on the side of too little (Fig 10C). Manual segmentation is biased toward higher variance since pixels are selected on a semantic basis (morphological structures the operator recognizes). Conversely, machine segmentation is biased toward lower variance since pixels are selected syntactically on the basis of pixel intensity distribution. Obviously, on the surface, literal tracing of the morphology is better, however this advantage can easily be lost in time constraints, noise, and optical depth. Notwithstanding these caveats, the point of segmentation is not accurate assessment of absolute intensity. Rather, the objective is more limited. We wish to accurately represent drug uptake ranking among samples and correctly distinguish cohorts which display statistical differences. In this more limited objective, we want to be assured that the machine is not adding systemic bias to predispose ranking or statistical interpretation different than from what would be obtained with manual segmentation, which we used as our validation standard.

Segmentation of cell cytoplasm, compared to segmentation of the heterogeneous shapes in typical photographic images, is a relatively simple task. We segmented on the basis of one color (however, the map algebra approach could be easily extended to two or more colors). In our research, the cell is segmented into 3 parts, the RoI, and two flanking regions ‘below’ and ‘above’ the pixel intensities in the RoI. Our primary goal was to segment the cytoplasm of intermediate cells in confocal images of the stria vascularis. In these images, the areas ‘below’ and ‘above’ are relative to the mean cytoplasmic pixel intensity. Intermediate cells are generally well-suited to machine segmentation since the images display sufficient difference in pixel intensity associated with morphology and cell type, e.g., intermediate cells and capillaries/endothelial cells. The ‘flatter’ pixel intensities of marginal and basal cells, as well as fibrocytes, were better characterized by the simple statistics from the gross segmented image.

It is important to note the mean is the ‘characteristic number’ in most statistical analyses, with pixel intensity the basis for calculating the mean. This does not imply, however, that pixel intensity is necessarily the best basis for demarcating the region of interest. Other variables such as the pixel intensity gradient, local autocorrelation, or degree of detected ‘edgeness’ could alternatively be used to demarcate regions. In practice, hybrids of general approaches are used, with “almost as many cell segmentation methods … as there exist cell analysis problems” [8]. The integrated pixel intensity methods we used primarily fall under the category of feature detection, principally for edges. Although the percentile method is not easily characterized in the Meijering schema [8], the intensity-based criteria of low and high quantiles function in a similar manner to morphological filtering. Local autocorrelation methods also involve masking, although the mask in this case is local autocorrelation. Only one Moran I parameter is needed since background and capillary neighborhood have similar local autocorrelation. We combined a masking image with local and global information to select pixels in the original image. In our images, this characterized the approximate location of the cell membrane as a starting point for subsequent algorithm development.

### Segmentation criteria for pixel intensity analysis

We identified four criteria. First, the method (or algorithm) should process all images in an image stacks without any additional user intervention. Second, the method should utilize local neighborhood information, so that isolated pixels incongruent with neighborhood values are discounted. Ideally, there should be facility for taking consideration of local information on higher order attributes, e.g. local autocorrelation. Third, the method should be robust within and across multiple image stacks (from an experimental series). This precludes methods contingent on substantial user input or supervision. Fourth, the method should be easily modulated, preferably via having easy access to settings that adjust the algorithm code.

Fiji (version 1.51n) has a number of segmentation algorithms [15], including algorithms based on merging regions [16] and training a classifier [17]. The region merging algorithm presents no obvious facility for processing an image stack. Trainable Weka segmentation, by contrast, produces good results on intermediate cell images and allows processing of a stack, however it is still deficient by a number of our criteria. The training classifier can be shared across images in the stack, however results from applying this single classifier of one image across a range of images are questionable. Another weakness by our criteria, the algorithm is not tweakable–we have only marginal access to the Java code. This is important for adjusting to the needs of intermediate cell images and to remedy any format incongruities. Other recent algorithms engendering excitement, though developed primarily for 3D segmentation, include ACME [18], RACE [19], and MIST [20, 21]. The strength of ACME is “quantitative analysis of cell dynamics during morphogenesis” [18], focusing on the tracking of cell membranes during development. These objectives are not well matched to our objective of assessing drug uptake. Likewise for RACE [19], with its primary use is in following cell shape, and tracking cell origin, during development. MIST [20] has its primary use in identifying structures from MRI images. It uses a promising approach, random-field based segmentation trained on manual segmentation results, and it primarily has been used to parse apart structures in the brain. The algorithm has also found use in material science applications [21]. The concerns we have with MIST are the similar to those we have with Trainable Weka segmentation. The classifier may not be generalizable to the range of confocal images used here.

### Integrated intensity-based methods

In our first attempts at solving the segmentation problem, we employed the first and second difference of the integrated pixel intensity. This approach is consistent with long standing gradient-based edge detection methods [22], the general idea to monitor a moving average and identify gradients of pixel intensity characterizing salient features of the morphology. We found first difference methods quite useable without empirical adjustments. We also were able to make second difference methods work, albeit with more difficulty. Part of the difficulty was in trying to utilize more information, e.g. utilize the ascending asymptote. The asymptote is quite noisy, and attempting to utilize the extra information made the algorithm cumbersome. However, second difference methods, which we tried first, led to the more tractable first difference methods. First difference methods gave a much cleaner morphology signal. The optimum of the first difference of the integrated pixel intensity was a good estimate of the cytoplasmic mean in our images of intermediate cells, with fixed interval pixel intensity thresholds to either side of this optimum giving a good estimate for delineating cytoplasmic areas for subsequent analyses. Integrated intensity methods were then superseded by percentile- and local autocorrelation-based methods.

### Percentile-based methods

The simplest and most elegant solution for pixel intensity analysis is percentile segmentation, and, on the surface, the effectiveness of simple percentile-based segmentation on our images is uncanny. Percentile based segmentation is little more than intensity-based thresholding, with percentile replacing pixel intensity. In this method, we set image pixel intensity thresholds to fixed percentiles of the original, grossly-segmented image, or to the masking smoothed image (Fig 5). The percentile curve is the inverse of the empirical cumulative density function (ecdf): the percentile gives the pixel intensity of a specific percentile, while the ecdf gives the percentile of a given pixel intensity. Correspondingly, because the percentile curve is the inverse of the integrated intensity curve, a connection is bridged with integrated intensity methods.

We made an implicit assumption that similarly-acquired images with a similar morphological structure display a similar percentile structure. This may account in part for the success of this method. The percentile structure reformats the pixel data. When formatted as percentiles the pixel data displays as sensitive and insensitive regions. Threshold changes in sensitive regions are associated with rapid changes in the percentile, while threshold changes in insensitive regions have little effect on the percentile (Fig 6B, 6D and 6F). Fixed percentiles were effective for segmentation because (1) most percentiles (corresponding to the pixel intensities along the *x*-axis) map to relatively insensitive regions, (2) images from the same imaging sessions tend to have similar sensitivity domains, and (3) the first difference of the vector of percentiles was consistently “U”-shaped, with the basin corresponding to the RoI (see Fig 6B, 6D and 6F). Deviations in thresholds from the insensitive pixel value regions (basin) did not affect the characteristic value of the RoI as much as deviations in threshold in sensitive pixel value regions. In other words, we found that it is more important that thresholds, however determined, lay in the correct sensitivity basin (accuracy) than thresholds are optimized, i.e., Fig 6. Changes in threshold in sensitive regions have substantially more effect on global statistics than changes in insensitive regions. Representing the data as percentiles increased the chance that estimated pixel intensity thresholds would lie in the range of the insensitive regions.

The most surprising result of the study was how well a simple percentile function performed in segmenting confocal images of cells that had taken up fluorescently-tagged gentamicin (gentamicin conjugated with Texas Red, GTTR). Simplicity is likely part of the reason, as we found simpler algorithms tended to be more robust than more complicated algorithms (probably due to the greater difficulty in clearing up boundary value problems in the complicated algorithms). Boundary value problems occur when the algorithm produces values that produce incommensurable values and the algorithm is unable to proceed further, crashing the program. As mentioned earlier, complicated second difference methods pose more difficult boundary value problems than first difference or percentile-based methods, and the former required more time to de-bug the programming script.

### Relation of integrated intensity and percentile methods to local autocorrelation

We have argued that local autocorrelation underlies other algorithmic approaches. Indeed, effective segmentation is a form of clustering and local autocorrelation. Morphological structures are effectively clusters, and with proper transformation, these clusters become visible. The integrated intensity- and percentile-based transformations vectorize the image into a mathematical construct, and ultimately throw away 2-dimensional spatial information. First and second difference methods based on intensity transformations are calculations made from histograms of the integrated intensity data, and thus, a step removed from the local morphology seen in the image. Integrated intensity-based methods can identify regions of pixel intensity space undergoing change relative to more stable pixel intensity regions. The pattern is detected in an integrated intensity transformed space, and not in the space of the original image. Despite its indirectness, this method was still effective in identifying homogeneous regions. Percentile-based segmentation appears to work similarly, although the method is much simpler. Jettisoned is the need for integrated intensity calculation, or for first or second difference optimization. Instead, the method relies on *a priori* assumptions. The image is assumed essentially tripartite, with lower and upper percentile thresholds set to pare away background and more-saturated pixels. The ‘focusing’ of the algorithm is entailed in the variability of the pixel frequency relative to the percentile thresholds. When pixel intensities are ‘bunched up’, as in homogeneous intensity intervals, the corresponding intensity thresholds tend to move outward towards the 0 or 1 extrema (since percentiles are based on pixel count). This expands or contracts the size of the segmented RoI. The implicit assumption is that heterogeneous regions are capped by homogeneous extrema.

The first difference of the percentile provided insight into how percentile-based segmentation is effective. The percentile is the inverse of the empirical cumulative density function. Rate of change in the percentile vector is a particularly intuitive way of visualizing plateaus of heterogeneous image brightness (as shown in Fig 6B, 6D and 6F). The visual patterns in an image are largely due to regions of similarity, and these, as reflected in the percentile gradient, were readily pulled out. One can consider cell membranes in the image as transition zones and the cytoplasm as plateaus. Both show characteristic patterns in the rate of change of the percentile. Thus, segmentation identifies salient regions of interest by locating the thresholds of the appropriate basin. Empirically, pixels framing a basin were more strongly autocorrelated (pixel values are clustered closer together) than pixels in the basin. Local autocorrelation enabled the localization of differences in the autocorrelation. Because of this differential in autocorrelation, small deviations in pixel intensity within the basin became relatively unimportant, and had relatively little effect on a characteristic value such as the mean or median. In other words, the first difference of the percentile remained relatively flat when stepped through pixel intensity within the basin. However, for the pixels framing the basin, the speed of change in the number of pixels per unit of intensity increased, generating a clear indicator of morphological change. By detecting the rate of change in the percentile vector we can easily optimize a characteristic value to identify the RoI and set lower and upper thresholds to circumscribe the basin.

Implicit in the discussion above is the fact images can also be segmented directly by local autocorrelation. Background, blood vessel walls, and nuclei cluster as higher values of the local Moran autocorrelation, and with appropriate setting of the Moran threshold, these features are selectively isolated. Local autocorrelation measures the homogeneity in the neighborhood of individual pixels, and this reflects different characteristics at different scales. When the correlation length is short, the homogeneity tends to reflect the optical resolution. When the correlation length is long, the homogeneity reflects morphology. In all cases, the homogeneity reflected in local autocorrelation is relative, not absolute. In this context local autocorrelation and integrated intensity are not totally independent of one another, as local autocorrelation often parallels the rate of change in the integrated pixel intensity. Since the local autocorrelation for both background and pixel intensity saturated regions tends to be high, one number (specifying degree of local Moran autocorrelation) was sufficient to demarcate between background/saturated regions and cytoplasm.

### Supervision and evaluation

We expect machine segmentation to give consistent results on proximate images and give results in concordance with manual segmentation results. While some discrepancy between machine and manual results is inevitable, the law of large numbers diminishes these discrepancies. As expected, we found robustness across *z*-stacks and to perturbation in percentile-segmented sets, and parallel trends in results across sets of manual- and machine-segmented images. Our test samples were restricted to one cell type, intermediate cells. In visual tests of these segmented cells, we found percentile methods notably more robust than integral pixel intensity methods. The integral intensity-based methods would on occasion produce a dramatic change in the segmentation as we stepped through the z-stack. Percentile methods, by contrast, produced the continuity we expected. We assessed the robustness of percentile segmented images to additive and multiplicative perturbation (Fig 9), and the results were stable over a wide range. We assessed the trend of manual and percentile segmented image results (mean of segmented regions) by cosine similarity, and found that although there was bias, the bias was fairly constant and in the same direction (Fig 10A). We compared the mean and variance of manual- and machine-segmented images, and found that manual segmented images consistently displayed higher variance (Fig 10C). Most importantly, we compared experimental results to assure distinctions in manually-segmented sets were replicated in machine-segmented sets. Although we found a fairly consistent offset between manual- and machine-segmented means, the experimental results from comparison of cohorts of manual- and machine-segmented sets were consistent. These evaluation results demonstrated that machine segmented images display sufficient consistency with other ‘adjacent’ images and sufficient concordance with manual segmentation results to be useful.

## Conclusions

We developed integrated intensity-, percentile-, and local autocorrelation-based algorithms to segment images of Texas Red-conjugated gentamicin uptake in inner ear cells. Manual segmentation was considered the gold standard for validating machine-based segmentation. A surprising result was that mathematical optimization is often not necessary. Simpler methods were more stable and easier to set up than more complicated methods (first and second difference methods). The simpler methods inevitably have simpler boundary conditions, and minimized the time needed to troubleshoot algorithms. In addition, we also found that methods tended to simplify with experience, as unneeded sophistication is eliminated under the selective pressure of supervision. The non-optimization based, simpler methods include local autocorrelation- or fixed percentile-based methods. Both simplified methods would be characterized as examples of morphological filtering in Meijering’s schema [8], ultilizing a masking image for selecting pixels in the original image. While the simpler methods rely more heavily on implicit assumptions, we found that, with experience, these concerns were often theoretical. In fact, the less-complex algorithms were more robust, and that machine-segmented images displayed less variance. Evaluation and validation demonstrated that machine-segmented images display sufficient consistency and concordance with manual segmentation to be a valid methodology for estimating mean pixel intensity in segmented images.

## Supporting information

S1 Data Scripts for machine segmentation and validation. (PDF)

S1 Fig Flow chart for integrated pixel intensity-based segmentation to obtain the first difference. (PDF)

S2 Fig Flow chart for integrated pixel intensity-based segmentation to obtain the second difference. (PDF)

S3 Fig Flow chart for local auto-correlation-based segmentation. (PDF)

S4 Fig Outcomes of manual- and machine-segmentation across experimental conditions. (**A**) When images for individual experimental groups were analyzed following manual segmentation, the intensity of biotin fluorescence in intermediate cells was significantly higher in mice treated with *Diphtheria* toxin (DT) than in mice treated with PBS. Thus, DT significantly increased the strial uptake of biotin in both transgenic mice expressing the DT receptor (DTR; *p* = 0.027) and wildtype (WT; *p* = 0.048) mice, when using manual segmentation, suggestive of an inflammatory response. (**B**) When images were analyzed following machine segmentation, the intensity of biotin fluorescence in intermediate cells was significantly greater (by Student’s t-test) in DTR mice treated with DT than in DTR mice treated with PBS (*p* = 0.021). The probability that wildtype (WT) tissues treated with DT were significantly different from control mice trended towards significance (*p* = 0.072), only slightly different from that obtained with manual segmentation in A (i.e., *p* = 0.048). Nonetheless, both machine and manual segmentation methods gave similar results.
(PDF)
