# Supplementary material for: Simplified, automated methods for assessing pixel intensities of fluorescently-tagged drugs in cells
Source: PLoS One. 2018 Nov 1;13(11):e0206628. doi: 10.1371/journal.pone.0206628 (PMC6211712; doi:10.1371/journal.pone.0206628)
Supplement: S1 Data — (PDF) [file pone.0206628.s001.pdf]

S1 Data. Scripts for machine segmentation and validation. We developed scripts for machine segmentation and validated their utility relative to manual segmentation. A set of images of intermediate cells from the cochlear stria vascularis were segmented (i) manually (visually) and (ii) by machine (computer) using algorithms. The images used in this manuscript are available at this webpage in the folder labeled "**Images for segmentation**", separated into subfolders as follows:

a) **Original** (raw data)
b) **Fine** (manually-segmented images)
c) **Gross** (segmented images prepared for machine segmentation), and
d) **Percentile-segmented** (machine-segmented images).

The R software for machine segmentation is in the "**Software**" folder. The text in the PowerPoint documents in this folder can be copied and pasted into R for running. There are 4 scripts: the long version and three shorter scripts: one for percentile stacks, an autocorrelation slider, and one for calculating simple statistics on stacks. In this folder, there is also PDF documentation for the longer segmentation script.

Expanded information:

Expanded information on how the manuscript figures were obtained are in each of the figure sub-folders within the folder "**Figures**". Algorithms written in R accompany each set of supplemental data figures, including the parameters set for type of segmentation.

The folders "**Figure 2**" to "**Figure 9**" contain files for each figure including:

1. the original image(s),
2. the segmented image(s),
3. the R script(s) for running the algorithm, and
4. a synopsis of the long version of the program (short versions for image stacks also require desktop folders for uploading files and downloading data).

In addition, each figure folder may contain:

1. graphs of the first and second difference,
2. histograms,
3. graphs of quantile change,
4. text file of expanded results, and
5. synopsis of results in an Excel file format.

The folder "**Figure 10**" contains statistical validation files, including an Excel file of the original data for Figure 10.
