# Supplementary material for: Simplified, automated methods for assessing pixel intensities of fluorescently-tagged drugs in cells
Source: PLoS One. 2018 Nov 1;13(11):e0206628. doi: 10.1371/journal.pone.0206628 (PMC6211712; doi:10.1371/journal.pone.0206628)
Supplement: S1 Fig — (PDF) [file pone.0206628.s002.pdf]

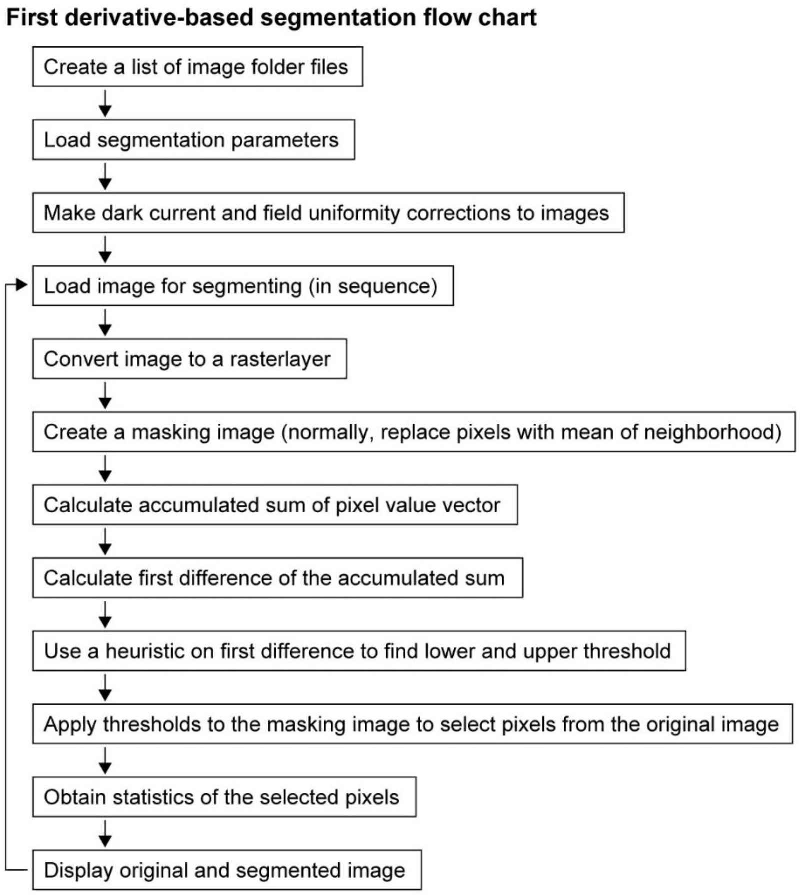

S1 Fig. Flow chart for integrated pixel intensity-based segmentation to obtain the first difference.
