# Supplementary material for: Simplified, automated methods for assessing pixel intensities of fluorescently-tagged drugs in cells
Source: PLoS One. 2018 Nov 1;13(11):e0206628. doi: 10.1371/journal.pone.0206628 (PMC6211712; doi:10.1371/journal.pone.0206628)
Supplement: S3 Fig — (PDF) [file pone.0206628.s004.pdf]

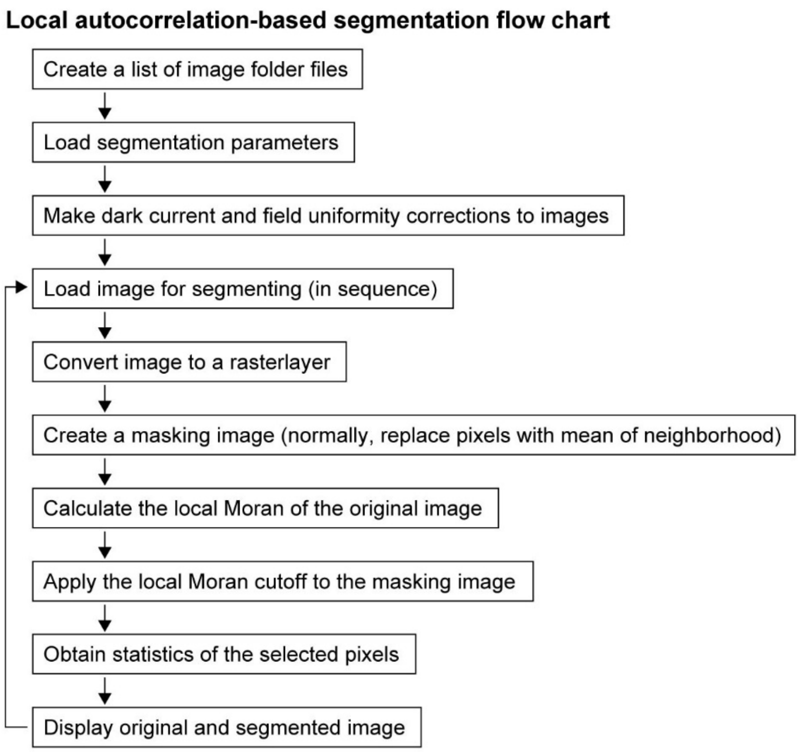

S3 Fig. Flow chart for local auto-correlation-based segmentation.
