# Supplementary material for: Simplified, automated methods for assessing pixel intensities of fluorescently-tagged drugs in cells
Source: PLoS One. 2018 Nov 1;13(11):e0206628. doi: 10.1371/journal.pone.0206628 (PMC6211712; doi:10.1371/journal.pone.0206628)
Supplement: S4 Fig — (A) When images for individual experimental groups were analyzed following manual segmentation, the intensity of biotin fluorescence in intermediate cells was significantly higher in mice treated with Diphtheria toxin (DT) than in mice treated with PBS. Thus, DT significantly increased the strial uptake of biotin in both transgenic mice expressing the DT receptor (DTR; p = 0.027) and wildtype (WT; p = 0.048) mice, when using manual segmentation, suggestive of an inflammatory response. (B) When images were analyzed following machine segmentation, the intensity of biotin fluorescence in intermediate cells was significantly greater (by Student’s t-test) in DTR mice treated with DT than in DTR mice treated with PBS (p = 0.021). The probability that wildtype (WT) tissues treated with DT were significantly different from control mice trended towards significance (p = 0.072), only slightly different from that obtained with manual segmentation in A (i.e., p = 0.048). Nonetheless, both machine and manual segmentation methods gave similar results. (PDF) [file pone.0206628.s005.pdf]

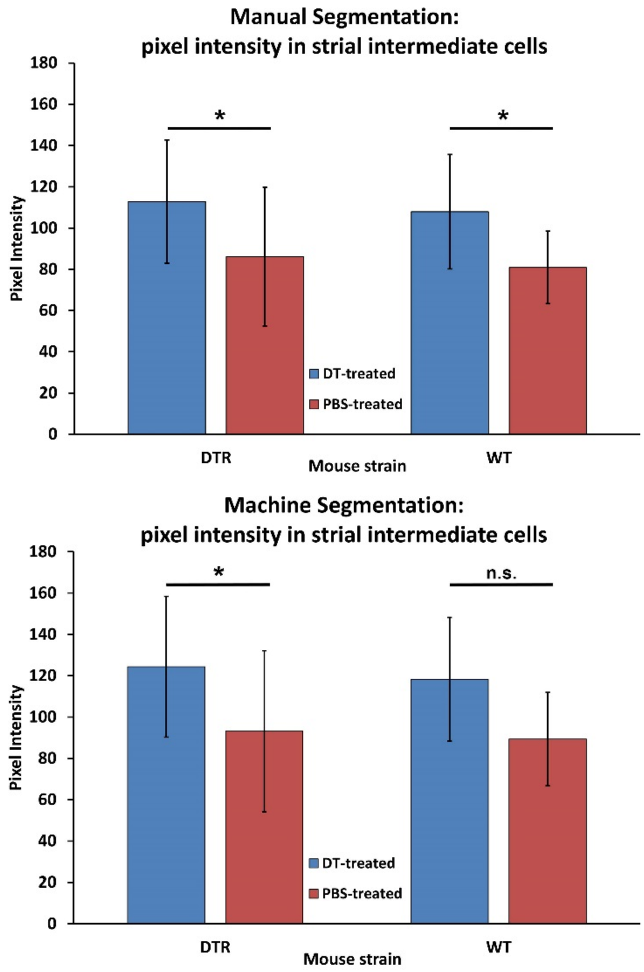

S4 Fig. Outcomes of manual- and machine-segmentation across experimental conditions (**A**) When images for individual experimental groups were analyzed following manual segmentation, the intensity of biotin fluorescence in intermediate cells was significantly higher in mice treated with *Diphtheria* toxin (DT) than in mice treated with PBS. Thus, DT significantly increased the strial uptake of biotin in both transgenic mice expressing the DT receptor (DTR; p=0.027) and wildtype (WT; p=0.048) mice, when using manual segmentation, suggestive of an inflammatory response. (**B**) When images were analyzed following machine segmentation, the intensity of biotin fluorescence in intermediate cells was significantly greater (by Student's t-test) in DTR mice treated with DT than in DTR mice treated with PBS (p=0.021). The probability that wildtype (WT) tissues treated with DT were significantly different from control mice trended towards significance (p=0.072), only slightly different from that obtained with manual segmentation in A (i.e., p=0.048). Nonetheless, both machine and manual segmentation methods gave similar results.
